# The Cerebellum and Psychiatric Disorders

**DOI:** 10.3389/fpubh.2015.00066

**Published:** 2015-05-05

**Authors:** Joseph R. Phillips, Doaa H. Hewedi, Abeer M. Eissa, Ahmed A. Moustafa

**Affiliations:** ^1^School of Social Sciences and Psychology, University of Western Sydney, Sydney, NSW, Australia; ^2^Psychogeriatric Research Center, Institute of Psychiatry, Faculty of Medicine, Ain Shams University, Cairo, Egypt; ^3^Marcs Institute for Brain and Behaviour, University of Western Sydney, Sydney, NSW, Australia; ^4^Department of Veterans Affairs, New Jersey Health Care System, East Orange, NJ, USA

**Keywords:** cerebellum, psychiatric disorders, cognitive processes, motor processes

## Abstract

The cerebellum has been considered for a long time to play a role solely in motor coordination. However, studies over the past two decades have shown that the cerebellum also plays a key role in many motor, cognitive, and emotional processes. In addition, studies have also shown that the cerebellum is implicated in many psychiatric disorders including attention deficit hyperactivity disorder, autism spectrum disorders, schizophrenia, bipolar disorder, major depressive disorder, and anxiety disorders. In this review, we discuss existing studies reporting cerebellar dysfunction in various psychiatric disorders. We will also discuss future directions for studies linking the cerebellum to psychiatric disorders.

The primary role of the cerebellum has traditionally thought to comprise balance and motor control. However, studies have been emerging that support multiple functions of the cerebellum including emotion regulation, inhibiting impulsive decision making, attention, and working memory (1–5). In addition, many experimental and computational studies show that the cerebellum plays a role in errorless (unsupervised) learning (6–8).

It has been suggested that motor (9), cognitive (10), and emotional abnormalities (5) can result from damage to parts of the cerebellum projecting to the motor areas, the prefrontal cortex, and the limbic system, respectively. Some further suggest that the cerebellar role in cognitive functioning is similar to the cerebellar control of purposive motor skills during motor functioning (11). There is also evidence that the cerebellum may be related to a variety of cognitive abnormalities and psychopathological manifestations (12). Many recent studies have reported a strong association between the structural and functional abnormalities of the cerebellum and psychiatric disorders especially schizophrenia (13, 14), bipolar disorder (15, 16), depression (17–20), anxiety disorders (21–23), attention deficit hyperactivity disorder (ADHD) (24–26), and autism (27, 28).

## The Cerebellar Circuits

The cerebellum communicates and influences information processing in multiple regions of the brain, including the cerebral cortex (29), spinal cord (30), vestibular nuclei (31), and the brainstem (e.g., the inferior olive and pontine nuclei) (32). Inputs from the spinal cord and brainstem enter the cerebellum through the inferior cerebellar peduncle. Also, afferents from the cerebral cortex (relayed in the pontine nuclei) enter through the middle cerebellar peduncle, and play a role in balance and movement (33).

The cerebellum projects to the brainstem and cerebral motor cortex via the red nucleus and ventrolateral nucleus of the thalamus (34). There are three output pathways from the cerebellum: (1) the cerebellar vermis indirectly to the pons, medulla, and reticular formation; (2) the intermediate zone of the cerebellum indirectly to the red nucleus and thalamus; and (3) the lateral zone of cerebellar hemisphere indirectly to the thalamus (35). After the thalamic connection, those fibers are projected to different parts of the cerebral cortex, including frontal cortex, motor cortex, and parietal cortex (35, 36).

**Figure 1:**
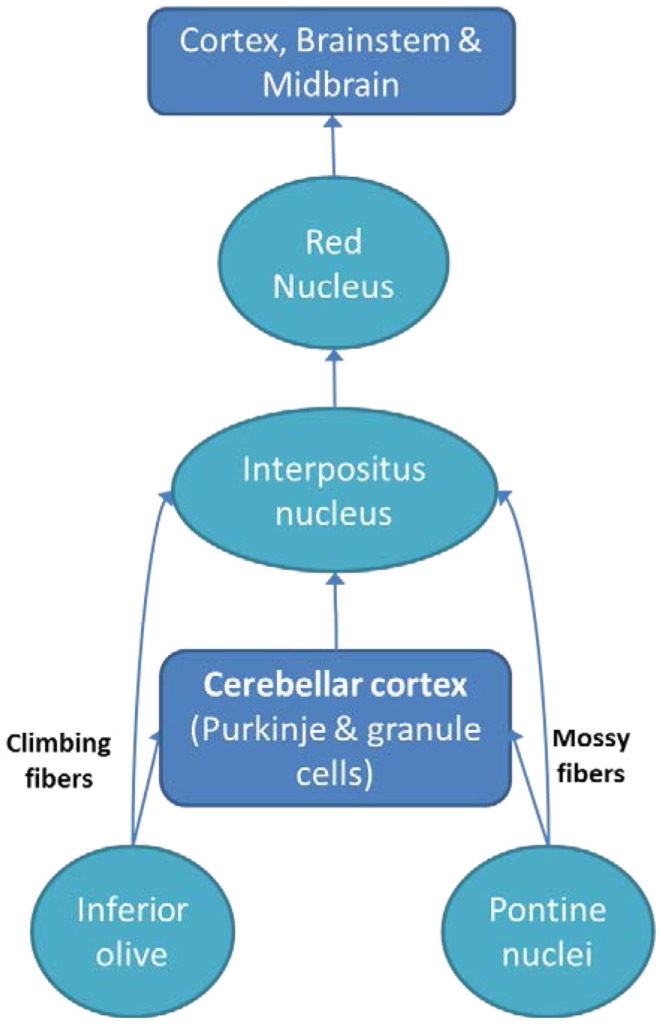
**A simplified diagram of the cerebellum along with connections with brain regions (cortex and brainstem)**.

The cortico-ponto-cerebellar and cerebello-thalamo-cortical pathways allow the cerebellum to affect information processing in cortical areas responsible for cognitive and emotional processes (2). These intricate connections between the cerebellum and other structures can explain why cerebellar damage can lead to various psychiatric disorders. Below, we discuss common psychiatric disorders associated with cerebellar abnormalities (see Figure 1 for a simplified cerebellar interactions with other brain regions).

## Attention Deficit Hyperactivity Disorder

Many studies report about 5% of children and adolescents aged 6–17 years are diagnosed with ADHD, while 30–50% of these individuals will continue to show ADHD symptoms into adulthood (2, 37). The diagnostic criteria of ADHD include three groups of symptoms: (1) attention deficit (easy distractibility, difficulty in concentration), (2) impulsiveness (impatience, negligence, impetuosity, difficulty in postponing answers, and rewards), and (3) hyperactivity (restlessness, agitation, excessive locomotor activity) (38). These groups of symptoms may be attributed to noradrenergic and/or dopaminergic neurotransmission dysfunction (39). Other theories about ADHD suggest a dysfunction to the frontal–subcortical pathway (40), while structural and functional neuroimaging studies show changes in prefrontal cortex, cingulum, basal ganglia, corpus callosum, and cerebral total volume (41–44). Multiple studies have also reported cerebellar changes in ADHD (17, 41, 45).

Until now little is known about how the brain develops in ADHD patients during the course of the disorder. Castellanos et al. (46) scanned adolescents diagnosed with ADHD (age 15–18) as well as healthy controls, to measure longitudinal changes (over a decade) of brain anatomy and volume. They found volumetric abnormalities with reduced cerebrum and cerebellum size that increased with age, while changes in the caudate nucleus volume disappeared as the subjects got older. These results were found to be unrelated to psychostimulant treatments (46). However, Ivanov et al. (45) found that patients undergoing stimulant treatment have larger overall cerebellar volume than untreated ADHD patients. This difference between treated and untreated patients may reflect the therapeutic mechanisms behind the stimulant treatment. The opposing results between the Castellanos et al. (46) study and Ivanov et al. (45) study may be due to the differences in the focus areas used by each study. Where Ivanov et al. broke down the cerebellum into its smaller regions, Castellanos et al. reported the volume change of the cerebellum as a whole, resulting in the loss of resolution of their data. Mackie et al. (47) conducted a longitudinal study comparing cerebellar differences between children with ADHD and healthy controls over the period of 2–14 years. ADHD patients were found to have smaller vermis than controls, which did not change with development. Vermis size could also predict the outcome for the patient, where smaller superior vermis volumes predicted poorer outcomes. Additionally, patients with smaller vermis lobules due to stroke or other developmental abnormalities also demonstrate a diminished attention-orienting ability (35, 48).

In sum, reduced cerebellar volume is a prevalent theme across studies investigating cerebellar abnormalities and ADHD. However, to date, these studies have only scanned and tested participants once they have been diagnosed with ADHD. This means that we are unable to determine if the abnormalities in the cerebellum were present from birth or if they developed during the child’s growth, and how this affects the etiology of ADHD. There are numerous longitudinal studies that recruit participants from birth or earlier. If these studies were to implement brain imaging at a young age, we may have a better understanding of how the cerebellum develops and whether there are any structural markers that predict the onset of ADHD in later childhood.

## Autism Spectrum Disorders

Autism spectrum disorder (ASD) includes a range of motor symptoms, including repeated and stereotyped movements, impaired social interactions [poor recognition of emotions, difficulty displaying physical gestures typically used in social interaction; (38)]. Interestingly, it was found that cerebellar damage in infants can predict the occurrence of autism in older age (49). The cerebellum is able to influence the motor cortex and prefrontal cortex area, two areas that are responsible for motor control and social cognition, so it is not surprising that abnormalities in the cerebellum would cause symptoms that observed in ASD.

Using a mouse model, Tsai et al. (50) have demonstrated in mutant mice that a decrease in Purkinje cell functioning leads to ASD-like behaviors, including abnormal social and motor behaviors (50, 51). This finding appears to be consistent with human studies as postmortem investigations have also shown a decrease in Purkinje cell density in patients with ASD (51, 52). Being GABAergic, a reduction of these cells may increase activity in the cerebellum–cortex pathway, which may explain the occurrence of repeated movements in ASD. This, however, needs to be confirmed or disconfirmed in future experimental studies that relate Purkinje cell loss to exact symptom domains (motor vs. social dysfunction) in ASD.

Using diffusion tensor magnetic resonance tractography, one study found altered connectivity in the superior peduncles and the short intra-cerebellar fibers in patients with Asperger’s syndrome [a mild disorder of the autism spectrum; (53)]. Decreased activity in the peduncle regions have also been related to poorer motor abilities in patients with ASD (54). There is an additional possible defect in the formation of cerebello-frontal circuits in Asperger’s syndrome (55). These deficits may be the cause of the motor and cognitive impairments observed in ASD-like patients.

Studies have also shown that impairment of adaptation of social behavior in patients with ASD may be caused by malfunctioning feedback pathways from the cerebellum to the cerebral cortex (56, 57). Also, the fibers of the middle and inferior cerebellar peduncles connecting the cerebellum with the frontal lobe are abnormally organized. This may be as either a direct cause or a consequence of changes in the cerebral cortex and cerebellar nuclei in patients with autism. Specifically, pathological changes are evident in the superior peduncles of the cerebellum in children with ASD. These pathological changes explain coordination deficits and ataxia, which are commonly presenting features in autistic-like behaviors (58).

Currently, there appear to be three main cerebellar abnormalities observed in patients with ASD: diminished Purkinje cells, reduced cerebellar volume, and interrupted feedback pathways between the cerebellar and cerebral areas. The latter two may also be bi-products of diminished Purkinje cells, suggesting that this is the root cause of the disorder. As Purkinje cells are inhibitory in nature, a lack of these cells would decrease inhibition that the cerebellum projects to the cortical and subcortical areas, leading to hypersensitivity of these brain regions found in most ASD patients (59).

Most studies to date on Purkinje cells and ASD focused on either Asperger’s syndrome or autism; however, it would be beneficial to investigate how Purkinje cell density is related to autistic severity. As Purkinje cells inhibit the cerebral cortex and mid-brain areas, we would surmise that patients with severe autism would also exhibit a much lower Purkinje cell density as they are more prone to being overwhelmed by stimuli. Additionally, if Purkinje cell density was to decrease further, the patient’s symptoms would worsen.

In sum, autistic spectrum disorders are developmental-based disorders; however, as studies focus on patients who have been diagnosed with the disorder, it is difficult to see when the neurological abnormalities began. Longitudinal studies beginning at birth that focus on functional and structural aspects of the child’s brain may offer predictive markers in the cerebellum that would increase the risk of developing ASD.

## Schizophrenia

Schizophrenia is a severe psychiatric disorder highly linked to genetic, environmental, and neurodevelopmental factors. Symptoms usually appear in late childhood and early adolescence and may include impaired thinking (delusions and hallucinations), disorganized speech, abnormal or catatonic behavior, and negative symptoms [e.g., avolition, flat affect, anhedonia; (38)]. It is estimated that the lifetime prevalence of patients with schizophrenia is about 1% of the general population (60). Cognitive deficits are also present in the disorder, demonstrated by an impairment of memory (61), learning (62), and executive function (63). Interestingly, many of the symptoms present in schizophrenia are similar to symptoms observed in patients with damage to the cerebellar cortex (64, 65).

Neuroimaging studies on schizophrenic patients have found that the cognitive deficits exhibited in some patients are related to cerebellar dysfunction, in particular, abnormal corticocerebellar connections (63, 66, 67). Many suggest that disturbances in the cortico-thalamic-cerebellar-cortical circuits play a role in cognitive functioning in schizophrenia. Moreover, Andreasen et al. (68) used functional neuroimaging to investigate brain activity in patients with schizophrenia while completing a memory recall task. They found a lower level of cortico-thalamic-cerebellar activity compared to healthy controls during task performance (68). It is not, however, known what kinds of functions are subserved by this pathway that could aid in cognitive performance.

Structural brain imaging studies have found reduced cerebellar volumes in schizophrenia patients, including diminished cerebellar vermis volume (69, 70). Changes in cerebellar volume in patients with schizophrenia have been linked to neural and behavioral abnormalities occurring in the perinatal period (71), male patients (72), onset at extremes of age (73), chronic nature of the disease (74), and clinical picture with predominantly positive symptoms (75).

Functional imaging studies in patients with schizophrenia reveal diminished blood flow to the cerebellar cortex and vermis during the performance of many cognitive tasks, such as attention, memory, including both short-term and working memory tasks (76), and social inference (77).

Studies regarding the role of the cerebellum in motor side effects seen in patients with schizophrenia on antipsychotic medications are limited. For example, one study showed a reduction in cerebellar activity in patients with schizophrenia developing akathesia during treatment with olanzapine (64); it is not, however, known, how changes to cerebellar function can lead to akathesia. Studies relating cerebellar function to treatment, or investigating cerebellar damage through the whole course of the disease and varying prognoses after using psychotherapeutic interventions are also scarce (78).

In sum, the current literature offers broad explanations of cerebellar abnormalities in schizophrenia, such as decreased volume, decreased blood flow, and dysfunctional cortical pathways. However, these features are also present in other disorders; for example, ASD and ADHD patients also exhibit a decrease in cerebellar volume. Smaller cerebellar volume in ASD can be attributed to decreased numbers of Purkinje cells; however, Purkinje cells do not differ between healthy controls and schizophrenia patients (79). This implies that cerebellar volume loss in schizophrenia is possibly due to the reduction or absence of different parts of the cerebellum. A closer look at which component of the cerebellum has depreciated in size or number will give a greater insight into the functioning of the cerebellum, and the role it plays in schizophrenia. Future research should also investigate whether there is difference between positive and negative symptoms and cerebellar functioning in schizophrenia. To our knowledge, there is only one study that found a relationship between cerebellar activation in schizophrenia and the occurrence of delusions (80).

## Bipolar Disorder

Bipolar disorder is characterized by alternating periods of mania and depression, with manic episodes lasting at least a week and depressive symptoms appearing immediately afterwards (38). Manic periods may involve abnormal thought patterns, euphoric moods, strong feelings of grandeur, hyperactivity, and impulsion, while depressive symptoms may consist of lack of motivation, psychomotor agitation, or retardation (38). The disorder may have an episodic course but more commonly, it is a chronic life lasting condition with a lifetime prevalence of 1.6% of the general population (81). The exact physiological and pathological mechanisms underlying bipolar disorder symptoms and the exact mode of action of mood stabilizers (including lithium) are not yet known. Many studies demonstrate cerebellar changes with decreased cerebellar volume and cerebellar atrophy in patients with bipolar disorder (15, 77, 82–84).

In review of studies comparing cerebellar volume in patients with bipolar disorder or major depressive disorder (MDD) with healthy controls, Soares and Mann (85) found smaller cerebellar regions present in both patient populations (85). It was not clear, however, how the reduction of cerebellar areas is related to disease progression or symptom severity. Interestingly, the volume of the V3 vermal subregion of the cerebellum is significantly reduced in multiple-episode bipolar disorder patients compared to healthy controls, while the volume of V2 vermal subregion is smaller in multiple-episode patients than first-episode patients (86). The strengths of the Mills et al. (86) study are the recruitment of different groups of bipolar patients as well as the investigation of subregions of the vermal region. Their results suggest that the severity of bipolar symptoms is associated with increased vermal damage. However, in a more recent study, bipolar patients does not show any significant differences in cerebellar volume compared to healthy controls (67). These contrasting findings may be due to the population tested by Laidi et al. (67). Participants were not controlled for their history of mediation, while it has been found that cerebellar volume reduction is much higher in medication naïve patients compared to patients undergoing anti-manic drug regime (87).

In a study using functional MRI in BD patients, increased glucose metabolism was found in the cerebellum of BD patients that were resistant to treatment (88). However, it is unclear whether these changes in cerebral blood flow and metabolism are primary or secondary to BD (89), which should be investigated in future studies. For example, it is not known whether these cerebellar changes are affected by treatment, as suggested by Ketter et al. (88). Testing both patients who are treatment-resistant and treatment-responsive and healthy controls can help understand the effects of bipolar treatment on cerebellar function.

In sum, there is currently contention in regards to the pathology of the cerebellum in BD. Laidi et al. (67) reported no difference in total cerebellar volume, while other studies report significant differences in cerebellar volume when compared to healthy controls (85–87). This difference is likely due to lack of controls over the participants (i.e., medication history). BD is also based on cycles between mania and depression; however, most studies do not take the patients current state into consideration during testing. Due to the inhibitory nature of the cerebellum, we would expect activation to decrease during manic phases, and increase during phases of depression. Alternatively, activation from the cerebellum could remain constant, while the rest of the brain is cycling while trying to compensate for the deviant inhibitory activation from the cerebellum. BD also has two manifestations: bipolar I and bipolar II sub groups. The difference between the two is that the latter involves manic phases that are less intense as those experienced in bipolar I. Investigating functioning and structural differences in cerebellum between the two subtypes may be able to isolate the manic component of the disorder, giving greater insight to the role the cerebellum plays on this aspect. To our knowledge, no study to date has investigated cerebellar structural or function difference between the two bipolar patient groups.

## Major Depressive Disorder

Patients diagnosed with MDD have experienced at least one depressive episode that may involve both motor and cognitive symptoms (38). Cognitive symptoms consist of difficulty concentrating or indecisiveness (38) are highly common and have been often linked to the prefrontal cortex and limbic system in MDD (90). In addition to these brain regions, patients with MDD have also shown various abnormalities in the cerebellum. Yucel et al. (91) found a significantly smaller vermis, an area responsible for the regulation of emotion and cognition (92), in MDD patients compared to healthy controls (91). Like bipolar disorder, studies also reported a smaller cerebellum in MDD patients (82).

Blood flow in the vermal areas of the cerebellum have also been linked to symptoms of MDD. Acutely depressed patients on various antidepressant medications showed an increased cerebellar activity and blood flow in the vermis when compared to remitting or healthy subjects. These findings were positively correlated with the severity of the depressive episodes, severity of cognitive deficits, and resistance to antidepressant medications (93–95). It is important to note that patients in the Liotti et al. study were not showing any depressive symptoms at the time of testing, thus suggesting that cerebellar activation patterns could reflect a trait marker for depression.

Further studies on medication naive patients also suggest abnormal cerebellar connectivity with the anterior cingulate cortex (19), an area known to influence affect, social functioning, motor control, and motivation (Paus, 2001). Abnormal connections between the cerebellum and frontal lobe have also been found in patients with severe depression and who are also resistant to treatment (5) and also reported in geriatric depression (96).

In sum, studies on the cerebellum and MDD have shown a reduced cerebellar size, an increase in cerebellar activity, and disrupted cortical connections. The reduction in cerebellar size is an interesting finding as this is also present in patients with ADHD. Additionally, this reduction for both patient groups appears to be focused on the vermis areas, an area that has been implicated in attention (35, 48), which is also impaired in patients with MDD (97). Interestingly, this area is also impaired in bipolar patients who exhibit attentional deficits (98). Further, although some studies investigated cerebellar activity in relation to severity of depressive symptoms (96), to our knowledge, no study has looked at the relationship between cerebellar function and individual symptoms in MDD, including anhedonia, low mood, or psychomotor retardation. However, some studies found that changes in cerebellar activity are not related to mood changes in MDD (99, 100).

## Anxiety Disorders

Anxiety disorders include disorders that involve excessive fear (concern about a current threat or perceive threat) and anxiety (concern about future threats or perceived threats). These disorders are typically coupled with extreme autonomic reactions, including muscle tension and elevated heart rate (38). The exact neural mechanisms underlying the occurrence of anxiety disorders are still unclear; some of the suggested mechanisms are decreased blood flow and metabolism in the frontal, temporal, parietal areas, and cingulate gyrus (101). In addition, impairment to the cerebellum has been reported in anxiety disorders and might be linked to increased arousal present in posttraumatic stress disorder (PTSD), generalized anxiety disorder (GAD) (102), and social anxiety disorder (SAD) (21).

Single photon emission computed tomography (SPECT) was utilized by Bonne et al. (103), which revealed increased cerebellar activity when re-experiencing the traumatic event in PTSD patients (103). In a study conducted on healthy subjects performing moderate exercise and complex mental arithmetic task, increased cerebellar and vermal activity was revealed in PET scanning. Cerebellar hyperactivity correlated positively with increased blood pressure and heart rate, highlighting a possible role for the cerebellum in the regulation of sympathetic activity, which may explain its role in anxiety disorders (104). These results were confirmed by another study on patients with panic disorder revealing a significant high-glucose metabolism levels in the pons, midbrain, medulla, thalamus, hippocampus, amygdala, and cerebellum (105).

In sum, most studies on anxiety and the cerebellum suggest a hyperactivity of the cerebellum; however, this is also true for patients with MDD. While this may be the cause of the attention impairments observed in both disorders, it would also be interesting to see which if any, areas are also contributing to the contrasting deficits that characterize each disorder. Comparisons of cerebellum activity during anxiety attacks with activity during a major depressive episode may help researchers understand how the role the cerebellum plays in each of these disorders. Data on role of treatment or psychotherapeutic interventions on cerebellar function are still unclear and warrant further studies. In addition, future comparative studies should also investigate cerebellar functions across anxiety disorders as well as symptom clusters in each anxiety disorder.

## Conclusion

Growing evidence and recent data suggest that the cerebellum plays a role not only in the control of balance and intentional voluntary movement but also plays an important role in the control of cognitive and emotional processes. The exact involvement of the cerebellum in these functions and its role in psychiatric and neurological disorders is clearly supported by functional and structural imaging studies. As discussed above, the cerebellum was found to be associated not only with psychiatric and cognitive symptoms in different disorders but also with pharmacological and behavioral therapies. However, it is still unclear how cerebellar dysfunction relates to different symptoms in psychiatric disorders. Future research using different motor and cognitive tasks in different types and subtypes of psychiatric and neurological disorders are still needed. Attention must be drawn to the interaction of genetic, developmental, structural, and functional brain changes involving the cerebellum in the production of symptoms in different psychiatric and neurological disorders.

The majority of studies are inconclusive when addressing specific anatomical abnormalities in the cerebellum that are present in psychiatric disorders. However, several of the disorders discussed share similar cerebellar abnormalities, for example, ASD, schizophrenia, bipolar, and MDD all show decreased volume in the vermis; however, their symptoms are remarkably different. As each area of the cerebellum projects to different areas of the cerebral cortex and mid-brain (106), the variety of symptoms suggests that the abnormalities of each disorder focused to specific areas, rather than the cerebellum as a whole. This may explain the wide range of symptoms observed across the disorders. For example, strong connectivity between the VIIb and IX vermis areas and the visual network has been noted by Sang et al. (106). This area is also known to have reduced blood flow in schizophrenic patients, which in turn could be a factor in visual hallucinations experience by the patient. The same can be said with hemispheric areas VI, VIIb, and VIII, which show connectivity with the auditory network (106) and could explain auditory hallucinations present in some schizophrenic patients. This problem highlights the need for more topographical studies focusing on smaller areas when looking for cerebellar abnormalities in these disorders.

In sum, our review shows that most prior studies of cerebellar function in psychiatric disorders did not focus on (a) investigating the different symptom domains for each disorder in relation to exact cerebellar damage, (b) testing which subregions of the cerebellum are related to the symptoms in each psychiatric disorder, (c) understanding drug effects, and (d) understanding neurodevelopmental changes associated with psychiatric disorders. In addition to experimental studies testing these points, theoretical analyses and computational modeling work are needed to explain how damage to certain subregions of the cerebellum relates to specific symptom clusters.

## Conflict of Interest Statement

The authors declare that the research was conducted in the absence of any commercial or financial relationships that could be construed as a potential conflict of interest.
